# Advanced chronic kidney disease with life-threatening hypokalemia due to undiagnosed Gitelman syndrome 

**DOI:** 10.5414/CNCS110977

**Published:** 2023-02-16

**Authors:** Artemios G. Karagiannidis, Maria-Eleni Alexandrou, George Lioulios, Maria Stangou, Pantelis A. Sarafidis, Aikaterini Papagianni

**Affiliations:** Department of Nephrology, Hippokration Hospital, Aristotle University of Thessaloniki, Thessaloniki, Greece

**Keywords:** Gitelman syndrome, hypokalemia, hypokalemic nephropathy

## Abstract

We report a case of a 58-year-old woman presenting with symptoms of oliguria, fatigue, anorexia, constipation, hypovolemic signs, and laboratory tests showing severe hypokalemia (1.7 mEq/L), hyponatremia (120 mEq/L), high serum creatinine (SCr, 6.46 mg/dL) and urea (352 mg/dL). The patient had previously been diagnosed with chronic kidney disease (CKD), with SCr up to 2.58 mg/dL 1 year prior, and had in all her previous laboratory tests shown hypokalemia, which was treated with conservative measures and eplerenone despite low-normal blood pressure and normal heart function. A set of coordinated measures were applied to restore the potassium deficit, revert hypovolemic hyponatremia, and support renal function (including 4 dialysis sessions). In addition, a careful diagnostic approach revealed inappropriately high urine sodium and potassium losses, hypocalciuria, and hyperreninemic hyperaldosteronism leading to the diagnosis of Gitelman syndrome and hypokalemia-associated chronic tubulointerstitial nephropathy. Importantly, compliance with a simple set of instructions on high potassium and liberal sodium diet enabled the patient not only to remain euvolemic, free of symptoms, and with normal electrolytes, but also to recover a significant part of renal function and stabilize at an earlier CKD stage. Gitelman syndrome is a rare disorder that can be easily diagnosed and treated following simple measures; its early diagnosis is necessary to avoid life-threatening complications.

## Introduction 

Gitelman syndrome (GS), also referred to as familial hypokalemia-hypomagnesemia, is a rare hereditary salt-losing tubulopathy. It is caused by several mutations, the most common of which concern the *SLC12A3* gene encoding the thiazide-sensitive NaCl cotransporter (NCC) in the distal convoluted tubule (DCT), and is inherited with the autosomal recessive pattern [1]. This apical cotransporter interferes exclusively with the NaCl reabsorption in the initial part of the DCT [2]. Typically, the syndrome includes a combination of electrolyte disorders resulting from impaired apical transport through the NCC, i.e., hypokalemia, metabolic alkalosis, hypomagnesemia, and hypocalciuria, which mimic the action of thiazide diuretics on NCC [3]. The clinical presentation of GS varies widely, while the long-term consequences of untreated disease are poorly defined. 

Herein, we describe a patient with chronic kidney disease (CKD) of unknown etiology presenting with dialysis-dependent renal dysfunction coupled with life-threatening hypokalemia and severe hyponatremia, which, after a careful diagnostic process was attributed to GS, enabling correction of electrolyte disturbances and sustained recovery of renal function. 

## Case report 

A 58-year-old White woman presented to the emergency department of our tertiary hospital complaining about oliguria, fatigue, and anorexia starting a few days ago, and constipation self-treated symptomatically by lactulose on a regular basis. Previous laboratory test results revealed CKD (serum creatinine (SCr) of 1.74 mg/dL 2.5 years prior, 2.58 mg/dL 13 months prior, 1.93 mg/dL 11 months prior, 1.98 mg/dL 5 months prior, and 5.12 mg/dL 1 week prior) with serum potassium and sodium of 3.39 mEq/L and 132 mEq/L 2.5 years prior, 2.37 mEq/L and 132.5 mEq/L 13 months prior, 2.31 mEq/L and 129.7 mEq/L 11 months prior, 3.47 mEq/L and 127.1 mEq/L 5 months prior, 1.79 mEq/L and 119 mEq/L 5 days prior, respectively. The rest of her past medical history included dyslipidemia, hypothyroidism, and β thalassemia trait. She was receiving eplerenone 25 mg once daily (o.d.), effervescent tablets of potassium bicarbonate 675 mg twice a day (b.i.d.), 25-hydroxycholecalciferol 400 IU o.d., calcium carbonate 500 mg o.d., epoetin alfa 5,000 IU subcutaneously every 3 weeks, folic acid 5 mg o.d., simvastatin 20 mg o.d., levothyroxine 25 μg o.d., and lactulose in large doses, especially during the last week. An extensive search in the patient’s past prescriptions via electronic records excluded the use of any other prescribed agent. 

On clinical examination, the patient’s temperature was 36.1 ^o^C, her blood pressure (BP) was 109/60 mmHg, her heart rate (HR) 60 beats/min, and her oxygen saturation (Sp0_2_) 98% in room-air. Heart and lung auscultation and abdomen clinical examination did not reveal any abnormal findings, but clinical signs of reduced intravascular volume (reduced skin turgor, cold extremities, and dry mucous membranes) were present. The remaining physical examination was unremarkable. 

Arterial blood gases analysis showed pH = 7.225, HCO_3_^– ^= 9.9 mEq/L, pCO_2 _= 24.9 mmHg, anion gap (AG) = 23.1 mEq/L, chloride (Cl^–^) = 86 mEq/L. Laboratory results, depicted in Table 1, showed elevated SCr (6.46 mg/dL) and urea (352 mg/dL), hematocrit of 22.6%, low serum potassium (1.7 mEq/L) and sodium levels (120 mEq/L) and high magnesium (4.8 mg/dL). Urinalysis was positive for protein and hemoglobin, while sediment showed 50-55 leukocytes and 51 – 80 red blood cells per high-power field. The electrocardiogram (ECG) revealed prominent U waves and prolonged QT interval (480 ms), whereas kidney ultrasound showed no evidence of obstruction and kidney size within normal with loss of corticomedullary differentiation. 

Based on the severity and urgency of clinical and laboratory findings, the patient received intravenous (IV) fluid therapy with 250 mL normal saline (N/S) enriched with 27 mEq of potassium chloride (KCl) within 2 hours at the short-stay unit of our hospital. Following that, the patient was admitted to the nephrology department for further investigation and treatment. Over the next days, the patient was treated with IV fluid administration with careful KCl supplementation (from 81 to 108 mEq/day) and careful bicarbonate administration. The patient’s diuresis was 700 mL within the first 8 hours and 1,200 mL during the next day. On day 2, not improving SCr (5.6 mg/dL) and urea levels (316 mg/dL) led to the initiation of hemodialysis (4 sessions in total) with high dialysate potassium. The patient also received transfusion with 2 units of red blood cells, and epoetin treatment was initiated. An antibiotic regimen with ciprofloxacin 200 mg b.i.d. was also initiated based on the positive urine culture. 

A 24-hour urine collection performed on day 2 showed excretion of creatinine at 485 mg/24h, urea at 7 g/24h, protein at 2.32 g/24h, sodium at 79 mEq/24h, and potassium at 8 mEq/24h, corresponding to a fractional excretion of potassium (FeK) of 42% and a urinary potassium-to-creatinine ratio (U_K _: U_C_) of 16.49 mEq/g. The patient received the last hemodialysis session on day 6; afterwards, SCr remained between 3 and 3.5 mg/dL and urea 80 – 100 mg/dL. Serum potassium reached normal levels (3.6 mEq/L) on day 10; on this day, urine potassium excretion was 34 mEq/24h, corresponding to a FeK of 27%, and indicating continuing potassium loss in the urine. Thereafter, the IV KCl supplementation was replaced with oral supplementation of potassium gluconate depending on the serum levels. 

On day 3, with the patient’s BP at 103/55 mmHg, plasma renin activity (PRA) was at 18.72 ng/mL/h (reference range in supine position: 0.2 – 1.4 ng/mL/h), and aldosterone at 36 ng/dL (reference range: 1.0 – 16 ng/dL), indicating heavy secondary hyperaldosteronism. On day 5, an echocardiogram revealed chamber sizes within normal range and normal systolic function (left ventricular ejection fraction of 60 – 65%), mild tricuspid and mitral valve regurgitation, mild aortic valve calcification, and a minimum amount of pericardial fluid located around the posterior heart wall. It has to be noted that the patient was previously referred to a cardiologist and a nephrologist and had, 3 and 4 years prior, undergone 2 PRA and aldosterone measurements, one time showing normal, and another time showing slightly elevated levels; she had also undergone a CT scan showing normal adrenal glands. 

After 13 days of hospitalization, the patient was discharged from the hospital with instructions to consume a high-potassium (two bananas daily) and liberal sodium diet as well as the following regimen: folic acid 5 mg o.d., calcium carbonate 500 mg 3 times a day (t.i.d.), potassium bicarbonate 1,350 mg b.i.d., levothyroxine 22 μg o.d., simvastatin 20 mg o.d., alfacalcidol 1 μg o.d., and epoetin alfa 5,000 IU subcutaneously 3 times a week. Four days later, the patient was evaluated in the nephrology clinic with SCr = 1.99 mg/dL and K^+ ^= 5.5 mEq/L, leading to potassium bicarbonate stop. The patient visited the clinic after ~ 1, 2, and 5 months; her laboratory results are presented in Table 1. As shown in the same Table, by following the instructions on high potassium and liberal sodium intake (80 – 90 mEq/day and 136 – 182 mEq/day), the patient was able to maintain euvolemia and normal serum potassium, be free of symptoms (dizziness, constipation, etc.), and regain a significant degree of renal function (SCr = 1.5 mg/dL). 

## Discussion 

GS is a common inherited tubulopathy, with heterozygote prevalence estimated at ~ 1% among Whites [4]. Loss-of-function mutations of NCC in early DCT cause reduced Na^+^ reabsorption, accompanied by isotonic water wasting. The resulting hypovolemic stimulus activates the renin-angiotensin-aldosterone system (secondary hyperaldosteronism). Increased Na^+^ delivery as well as aldosterone levels in the distal nephron lead to Na^+^ reabsorption in exchange of K^+^ (kaliuria) and H^+^ (aciduria) [5]. Increased magnesiuria and hypocalciuria usually also appear. Suspicion of GS is based on following criteria [3]: 1) chronic hypokalemia with inappropriate renal potassium wasting (increased FeK and U_K _: U_C_); 2) metabolic alkalosis; 3) hypomagnesemia with inappropriate renal magnesium wasting (increased FeMg); 4) hypocalciuria (reduced FeCa); 5) fractional excretion of chloride (FeCl) > 0.5%; 6) high PRA; 7) low or normal-low BP; 8) normal renal ultrasound [3]. 

Although some patients with GS may remain asymptomatic, most of them may present a variety of clinical symptoms and signs, including salt craving, fatigue, neuromuscular symptoms (dizziness, paresthesia, carpopedal spasms, muscle weakness, joint pains), renal symptoms (polyuria, polydipsia, nocturia), gastrointestinal complains (constipation, abdominal pain), cardiovascular symptoms (palpitations, ventricular arrhythmias), and developmental disturbances (short stature, growth and/or pubertal delay) [3, 6]. Of note, Fujimura et al. [7] showed that over 50% of GS patients are diagnosed through random blood tests, despite experiencing several symptoms for variable time. 

This case highlights important lessons regarding GS. Firstly, in our patient, the diagnosis was delayed for a long time, despite the existence of highly suggestive symptoms and persistent hypokalemia and hyponatremia for many years. The patient had previously been seen by physicians of different specialties, including general practitioners, a cardiologist, and even a nephrologist, all of whom ignored the above and treated hypokalemia symptomatically with potassium supplementation and initiation of eplerenone, probably due to mistaken interpretation of a normal PRA and aldosterone test and normal adrenal morphology. An increase in PRA and aldosterone might not have been detected though, if hypovolemia had been temporarily restored at that time point, allowing PRA and aldosterone to return to nearly normal levels. Secondly, this chronic hypokalemia led to a progressively reduced renal function, reaching SCr of 2.58 (equivalent to an estimated glomerular filtration rate (eGFR) of 20 mL/min/1.73m^2^, i.e., CKD stage 4) already 1 year before her hospitalization, with the patient being informed about the possibility of needing to begin renal replacement therapy in the long run. A superimposed acute kidney injury (AKI), possibly due to volume depletion, further reduced renal function and necessitated initiation of hemodialysis. However, the appropriate diagnosis of hypokalemia-associated chronic interstitial nephritis and proper care of the underlying GS with a straightforward set of in- and out-of-hospital measures enabled the patient to recover a large degree of renal function, reaching SCr of 1.5 mg/dL and eGFR of 38 mL/min/1.73m^2^. 

On admission to our clinic, the patient presented with hypovolemia, oliguria, severe hypokalemia, hyponatremia, hypochloremia, and partially compensated metabolic acidosis with increased AG, pointing towards the coexistence of hypochloremic metabolic alkalosis, possibly due to the hypovolemia. Based on the above, the working diagnosis of AKI superimposed on CKD, instead of progressive CKD to end-stage, was made. The emergency efforts were directed towards progressive restoration of hypokalemia, hypovolemic hyponatremia, and prerenal AKI. In addition, a parallel effort was made to reach a conclusive diagnosis of the underlying disease. 

**Figure 1.**
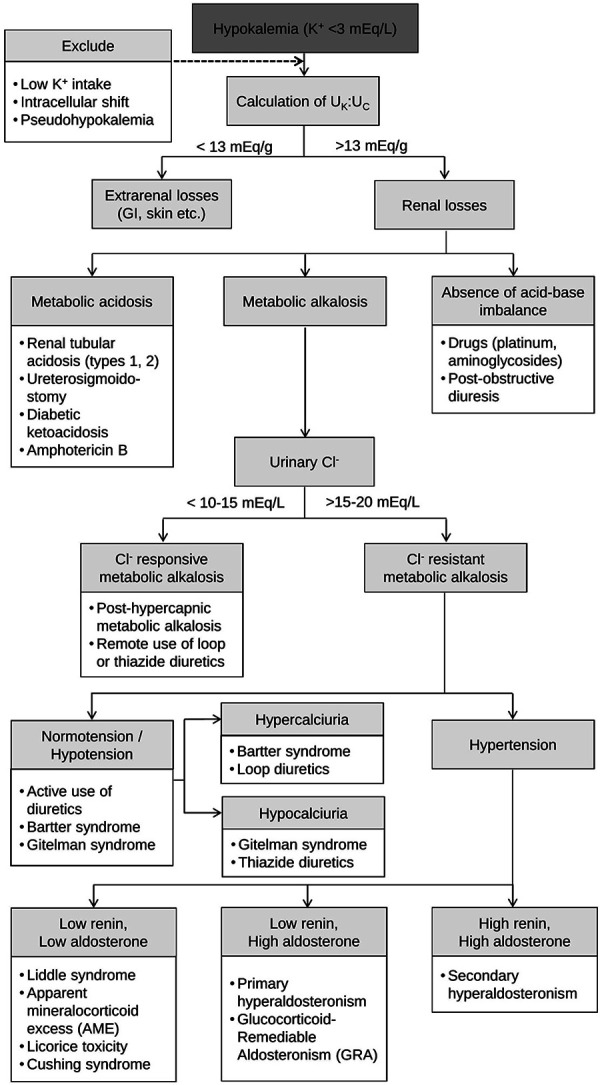
Diagnostic algorithm of hypokalemia focused on renal losses. K^+^ = potassium; U_K_:U_C_ = urinary potassium-to-creatinine ratio; GI = gastrointestinal; Cl^–^ = chloride.

Following continuous administration of isotonic solutions enriched with potassium, serum potassium levels were increasing slowly, a fact indicating the presence of chronic, severe total-body potassium deficit. In the case of our patient, an underlying salt-wasting tubular disorder, aggravated by intestinal losses due to lactulose overuse were possible. In any case, two calculations of FeK and U_K _: U_C_, both validated tools in assessment of hypokalemia in healthy and diseased populations [3, 8], confirmed inappropriate kaliuresis despite very low serum potassium. Results of high PRA and aldosterone were compatible with hyperreninemic hyperaldosteronism. These findings, together with the high urine chloride concentration (> 15 – 20 mEq/L), a urine calcium of 63 mg/24h (< 100 mg/24h), and the absence of use of thiazide diuretics, pointed towards the diagnosis of GS as the most plausible cause of our patient’s clinical presentation, according to existing diagnostic algorithms (Figure 1) [9, 10]. Our patient presented with hypermagnesemia, instead of the hypomagnesemia anticipated by the diagnostic features of GS. This finding could be explained either by reduced renal magnesium excretion in the presence of reduced renal function [11], or by overconsumption of lactulose, as this laxative can increase the intestinal absorption of Mg^2+^ when it is catabolized to organic acids in the large intestine [12]. 

**Figure 2.**
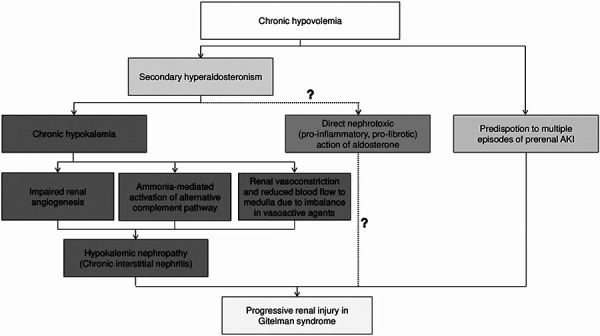
Pathophysiology of progressive renal injury in Gitelman syndrome. AKI = acute kidney injury.

Development of CKD in our patient with GS can be of multifactorial origin (Figure 2). Chronic hypokalemia per se causes tubulointerstitial nephritis with tubular vacuolization, cystic formations, and interstitial fibrosis in renal biopsy [13]. Suggested mechanisms include impaired renal angiogenesis and concomitant peritubular capillary loss linked with poor vascular endothelial growth factor (VEGF) expression [14], an imbalance in vasoactive agents (increased local endothelin-1 and angiotensin II, reduced renal kallikrein and NO) causing renal vasoconstriction and reduction of blood flow and oxygenation in renal medulla [15, 16], and increased local ammoniagenesis leading to activation of the alternative complement pathway [17]. Furthermore, chronic hypovolemia renders patients prone to experiencing multiple episodes of prerenal AKI, especially when adjacent factors coexist (e.g., dehydration, diarrheic episodes) [18]. Finally, the high levels of aldosterone incited by the chronic volume depletion could exert a direct nephrotoxic action, related to the expected pro-inflammatory and pro-fibrotic effects mediated by activation of mineralocorticoid receptors, as shown previously in models of AKI, hypertensive, and diabetic kidney disease and in cases of calcineurin-inhibitors toxicity [19]. This hypothesis was supported by an observational study of renal function in patients with GS and Bartter syndrome, which concluded that the degree of hypokalemia per se cannot be directly correlated with the degree of GFR impairment, and possibly aldosterone itself plays a major role in this impairment [20]. However, in a small study of 6 patients with GS, absence of the pro-inflammatory and pro-fibrotic effects of aldosterone, caused by increased oxidative stress and activation of Rho kinase pathway, was noted [21]. Thus, further research specifically in individuals of this type is needed. 

An interesting fact regarding our patient is that her renal function was constantly low for some years and improved considerably within a few months after the hospitalization. However, in some patients with tubulointerstitial disease, given that irreversible damage of chronic fibrotic tubulointerstitial injury is not extensive, withdrawal of the pathogenetic factor may not only stabilize, but also slightly improve renal function [22, 23]. In such cases, the presence of a portion of glomeruli without extensive damage may allow for increased filtration in the intact remained nephrons, which, according to traditional theory, could compensate, at least in part, for the function lost with the destroyed nephrons [24, 25]. To the best of our knowledge, this is the first case of a patient with hypokalemia-associated chronic tubulointerstitial nephropathy who impressively doubled their renal function within a few months of withdrawal of the pathogenetic factor. 

In conclusion, this case highlights the fact that failure of proper diagnosis of GS leading to chronic coexistence of hypovolemia and hypokalemia can lead to significant renal function loss requiring dialysis. In such cases, the impressively low serum potassium accompanying hypovolemia should always remind the clinicians of the possibility of a tubular disorder and trigger the relevant diagnostic work-up that can easily reveal the proper diagnosis. This can lead to institution of simple measures to correct relevant disturbances and most importantly, after the acute phase, to a proper guidance of the patient on simple dietary measures that can preserve normal electrolyte and fluid balance, resolve chronic symptoms, and result in maintenance, or even improvement, of renal function. 

## Funding 

This paper was not supported by any source and represents an original effort of the authors.

## Conflict of interest 

None declared. 

**Table 1.** Laboratory investigation results.

Variable	Day 1 admission	Day 2	Day 4	Day 11	Day 13 discharge	4 days later	11 days later	1 month later	2 months later	5 months later	Normal value or range
White cell count (10^3^/μL)	13.7	10.6	13.2	9.00	8.70	7.6	6.2	6.56	5.5	5.67	3.8 – 10.5
Hematocrit/hemoglobin (%, g/dL)	22.6/ 7.5	20.1/ 6.6	23/ 7.6	23.9/ 7.9	27.1/ 8.9	32.3/ 10.2	34.4/ 10.8	34.1/ 10.8	36.4/ 11.6	32.4/ 10.4	37.0 – 47.0 / 12.0 – 16.0
Platelets 10^3^/µL	331	314	269	342	351	489	433	306	336	282	150 – 450
Total proteins/albumin (g/dL)	6.1/ 3.8	5.1/ 3.3	5.2/ 3.3	–/–	5.0/ 3.1	5.8/ 3.6	5.7/ 3.7	–/–	–/4.0	–/–	6.6 – 8.3/ 3.4 – 5.2
Glucose (mg/dL)	117	158	155	–	149	81	86	–	–	89	70 – 125
Urea (mg/dL)	352	316	210	64	72	74	81	95	78	74	15 – 43
Creatinine (mg/dL)	6,46	5.6	5.11	2.48	2.33	1.99	1.74	1.69	1.76	1.5	0.66 – 1.1
Potassium (mEq/L)	1.7	2.2	2.7	3.6	4.1	5.5	4.6	3.86	4	3.75	3.5 – 5.1
Sodium (mEq/L)	120	127	134	141	141	136	138	–	137	137.1	136 – 145
Calcium (mg/dL)	10.8	9.0	9.3	–	7.0	7.9	9.3	–	9	8.97	8.5 – 10.5
Phosphorus (mg/dL)	–	10.4	5.9	–	3.9	3.5	3.8	–	4.1	–	2.5 – 4.5
Magnesium (mg/dL)	4.8	3.92	–	1.86	–	–	2.51	–	1.65	1.66	1.9 – 2.5
Uric acid (mg/dL)	8.2	8.1	–	–	5.7	5.8	6.4	–	6.8	6	2.6 – 6.6
Parathyroid hormone (pg/mL)	–	7.8	–	–	67.2	–	–	–	–	132	12 – 88
Urine volume	–	1,200	1,800	2,800	–	2,400	4,000	5,350	–	5,200	1,000 – 2,500 mL/24h
Urine creatinine	–	485.76	822.96	866	–	832.32	945.6	717	–	–	14 – 26 mg/24h/kg
Urine urea	–	7	9.3	6.5	–	11.5	14.1	–	–	–	20 – 35 g/24h
Urine total protein	–	2,323.2	1,409.4	–	–	–	440	524	–	496	< 150 mg/24h
Urine magnesium	–	–	90	–	–	–	–	–	–	–	73 – 122 mg/24h
Urine calcium	–	–	63	–	–	–	–	–	–	–	100 – 300 mg/24h
Urine sodium	–	79	124	168	–	130	168	182	–	136	50 – 200 mEq/24h
Urine potassium	–	8	14	34	–	62	52	80	–	90	30 – 90 mEq/24h
Urine chloride	–	–	140	–	–	–	–	–	–	–	110 – 250 mEq/24h
Urine phosphorus	–	–	36	–	–	–	–	–	–	–	400 – 1,300 mg/24h
Urine glucose	–	–	54	–	–	192	200	–	–	–	< 500 mg/24h
